# Selective DNA Delivery to Tumor Cells Using an Oligoarginine-LTVSPWY Peptide

**DOI:** 10.1371/journal.pone.0110632

**Published:** 2014-10-22

**Authors:** Cheng Gong, Deng Pan, Fengwu Qiu, Pei Sun, Yu-Hui Zhang

**Affiliations:** 1 Britton Chance Center for Biomedical Photonics, Wuhan National Laboratory for Optoelectronics-Huazhong University of Science and Technology (HUST), Wuhan, Hubei, China; 2 Key Laboratory of Biomedical Photonics of Ministry of Education, Department of Biomedical Engineering, Huazhong University of Science and Technology, Wuhan, Hubei, China; University of Pittsburgh School of Medicine, United States of America

## Abstract

DNA therapy for cancer requires efficient, selective and safe DNA delivery systems. Compared with other non-viral methods such as lipid or polymer-based DNA delivery vectors, peptide-based DNA delivery systems are biocompatible and biodegradable, which leads to lower immunogenicity and lower toxicity. Moreover, peptide vectors are easier to produce and their compositions easier to control because solid-phase peptide synthesis has been extensively developed. However, peptide-based systems for DNA delivery toward special tumor cells or tissues are still lacking. In this study, we constructed a non-viral 9rR-LTVSPWY peptide-based DNA delivery system and showed that it is able to efficiently and selectively transfect DNA into targeted tumor cells. This work presents a novel strategy for tumor cell-specific DNA delivery and a reference for designing more efficient DNA delivery systems targeted towards various types of cancer.

## Introduction

DNA therapy for cancer requires efficient and safe systems that can deliver the therapeutic DNA selectively into targeted tumor cells. Viral vectors have been used to transfer genes into cancer cells successfully [1]–[4]. However, these vectors have serious disadvantages, such as limited loading capacity, complexity of production, innate immunogenicity, and the risk of inflammatory responses and toxicity, that limit their clinical applications [5], [6]. To avoid these problems, various non-viral carriers have been developed; these carriers display low immunogenicity, relative safety, ease of production, and no cargo size limitation. Of the existing non-viral vectors, cationic lipids and cationic polymers are the most intensively studied and frequently employed. However, they have dose-dependent toxicities in *in* *vivo* applications [7], [8].

Cationic peptides have also been explored as gene delivery systems due to several advantages: biodegradability, biocompatibility, less toxicity, and ease of synthesis compared with polymeric carriers [9], [10]. Moreover, the composition of peptides is easy to control. By altering the composition of a given peptide, various functions can be achieved. Self-Assembling Peptides (SAPs) have been investigated as a tool for targeted tumor drug delivery based on the enhanced permeability and retention (EPR) method [11], [12]. However, EPR is not very effective, and the size dependency, slow time frame, variability from tumor to tumor and relative inability to operate in non-tumor vascular beds limit the clinical applications of the EPR method [13]. Furthermore, SAPs themselves usually cannot bind DNA; therefore, this method cannot be used as a DNA delivery system.

Cell-penetrating peptides (CPPs) are short peptides that can deliver cell-impermeable compounds into living cells and have been successfully employed to translocate various bulky cargos (including peptides, proteins, siRNA, DNA, and nanoparticles) across cellular plasma membranes [14], [15]. Among the CPPs, oligopeptides based on arginine are frequently used because of their ease of synthesis and cell-penetrating ability compared with other peptides based on other amino acids such as lysine [16]. With some modifications, cationic oligoarginines have successfully been employed to transport DNA into cells [17]–[19]. However, these systems lack selectivity toward the tumor cells, which is regarded as a prerequisite for safe and successful gene therapy [20]. Thus, developing an arginine-rich peptide that can mediate tumor cell-specific DNA delivery is very relevant for cancer gene therapy.

Targeted delivery of drugs into tumor cells using specific extracellular receptors has the following advantages in cancer therapy: (1) limiting adverse side effects caused by the drug absorption of normal cells; (2) enhancing drug internalization by tumor cells; (3) solving the resistance problem based on the active drug efflux from tumor cells [21]. LTVSPWY, a 7-residue peptide, has been shown to specifically bind to and be absorbed by certain types of cancer cells, possibly via receptor-mediated endocytosis [22]. Moreover, certain LTVSPWY-attached nanoparticles have successfully been absorbed by these tumor cells [23].

In this study, we explored the possibility of using an oligoarginine-LTVSPWY peptide as a non-viral vehicle to deliver DNA selectively into tumor cells. The peptide has a tri-block design composed of nona-arginine (rRrRrRrRr, r: d-Arg, R: l-Arg) for binding DNA through electrostatic interactions, four histidine residues as a spacer and for enhancing endosomal escape [10], and the LTVSPWY sequence, which is used for tumor cell targeting and cell adsorption.

## Materials and Methods

### Materials

The plasmid pEGFP-N1 was obtained from Clontech (CA, USA), and the pGL3 control vector was from Promega (WI, USA). The peptides 9rR-LTVSPWY (rRrRrRrRrHHHHLTVSPWY) and 9rR (rRrRrRrRr) [24] were prepared using solid-phase peptide synthesis and purified to homogeneity by preparative high performance liquid chromatography (HPLC) to achieve >95% purity. Their appropriate masses were confirmed by electrospray ionization (ESI) mass spectrometry (Figures S1 and S2).

### Cell culture

5–8F cells (a human nasopharyngeal cancer cell line) [25] were a kind gift from Prof. Yi-Xin Zeng and Prof. Mu-Sheng Zeng (Sun Yat-sen University Cancer Center, Guangzhou, China). SKOV-3 cells (a human ovarian cancer cell line) were from the American Type Culture Collection (ATCC). SKOV-3, MCF-7 and 5–8F cells were cultured in RPMI1640 supplemented with 10% fetal bovine serum (FBS). HeLa cells were cultured in Dulbecco’s modified Eagle’s medium (DMEM) containing 10% FBS. The cells were grown in a CO_2_ (5%, v/v) atmosphere at 37°C.

### Determination of zeta potential and particle size of complexes

The peptide/pDNA complexes were formed via self-assembly. The N/P ratios represent the molar ratio of the arginines in 9rR (or the arginines in the nona-arginine fragment of the 9rR-LTVSPWY) to the phosphorus content in the DNA [19]. The full procedure was performed as previously described [24].

### DNA migration assay

Peptide/pDNA complexes at the indicated N/P ratios were prepared in PBS (phosphate buffered saline), and the pDNA amount was kept constant (0.1 µg). Agarose gel electrophoresis (0.8%) was performed in Tris-acetate (TAE) running buffer at 150 V for 10 min.

#### Plasmid labeling

The procedure was performed as previously described [24]. 200 µg of pEGFP-N1 was incubated with 5 µg ethidium monoazide (EMA) in 2 mL of ddH_2_O for 10 min at room temperature. The solution was exposed to UV light for 5 min and then visible light for 10 min. The plasmid was precipitated by the addition of ethanol, collected by centrifugation at above 10,000 rpm for 15 min, and redissolved in ddH_2_O.

### Confocal microscopy

Cells were seeded into eight-well Lab-Tek chamber slides (Nunc) at a density of 1.0×10^4^ cells/well in 200 µL RPMI1640 (for SKOV-3, MCF-7 and 5–8F cells) or DMEM (for HeLa cells) supplemented with 10% FBS. The rest of the procedure was performed as previously described [24].

To track the internalization of the plasmids, 5–8F cells were incubated with the indicated peptide/EMA-labeled pEGFP-N1 complex (N/P 6∶1, 240 µL, 1 µg pDNA) and LysoTracker Green (100 nM, Invitrogen, USA) at 37°C or 4°C for 30 min. (For the 4°C experiments, the cells were pre-incubated at 4°C for 15 min). Then, the media was replaced with RPMI1640 containing 10% FBS. The rest of the procedure was performed as previously described [24]. The co-localization analysis was performed using the Manders Coefficients plugin of Image J.

### Flow cytometry

Cells were seeded into a 24-well plate at a density of 5.0×10^4^ cells/well in 400 µL RPMI1640 (for SKOV-3, MCF-7 and 5–8F cells) or DMEM (for HeLa cells) containing 10% FBS. Cells in the logarithmic phase were incubated with the indicated peptide/pEGFP-N1 complexes (480 µL, 2 µg pDNA) at the indicated N/P ratios in full serum medium without antibiotics for 5 h. Subsequently, the media was replaced by medium containing 10% FBS. The rest of the procedure was performed as previously described [24].

To track the intracellular uptake of the plasmids, 5–8F cells were incubated with the peptide/EMA-labeled pEGFP-N1 complexes (N/P 6∶1, 480 µL, 2 µg pDNA) at 37°C for 30 min. For the 4°C experiments, the cells were pre-incubated at 4°C for 15 min and then incubated with the complexes for 30 min. Subsequently, the media was replaced by RPMI1640 containing 10% FBS. The rest of the procedure was performed as previously described [24].

### Luciferase expression assay

Cells were seeded into a 96-well plate at a density of 5×10^3^ cells/well in 100 µL RPMI1640 (for SKOV-3, MCF-7 and 5–8F cells) or DMEM (for HeLa cells) containing 10% FBS. Cells in the logarithmic phase were incubated with the indicated peptide/pGL3 complexes (120 µL, 0.5 µg pDNA) at an N/P ratio of 6∶1 in full serum medium without antibiotics for 5 h. Subsequently, the media was replaced by media containing 10% FBS. The rest of the procedure was performed as previously described [24].

### Cell toxicity

The cytotoxicity of 9rR-LTVSPWY was evaluated using a 3-(4, 5-dimethylthiazol-2-yl)-2, 5-diphenyltetrazolium bromide (MTT) assay. 5–8F cells were incubated in a 96-well plate at a density of 5×10^3^ cells/well with the 9rR-LTVSPWY/pEGFP-N1 complex (N/P 6∶1, 120 µL, 0.5 µg pDNA). The rest of the procedure was performed as previously described [24].

## Results

### Characterization of 9rR-LTVSPWY/pDNA complexes

Our previous study has indicated that the replacement of l-Arg residues with d-Arg residues imparts greater proteolytic resistance of oligoarginine peptides [26]. We used a heterogeneous nona-arginine (rRrRrRrRr, r: d-Arg, R: l-Arg) peptide because the heterogeneous peptide is more protease resistant than the l-oligoarginine, which is more bioavailable. Additionally, the heterogeneous peptide is more biodegradable than the d-oligoarginine, leading to reduced toxicity [26]. An LTVSPWY segment was added into the peptide sequence as a tumor cell-targeting moiety.

**Figure 1:**
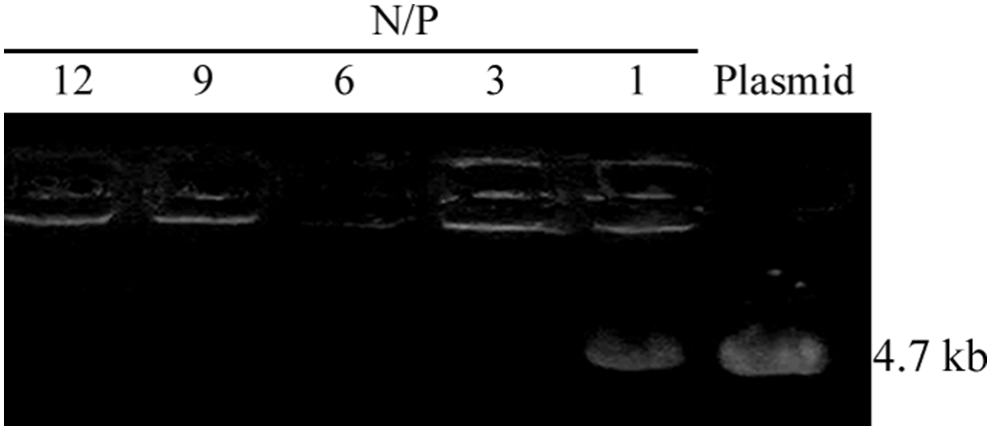
Agarose gel electrophoresis of the 9rR-LTVSPWY/pEGFP-N1 complexes at the indicated N/P ratios.

The formation of the 9rR-LTVSPWY/pDNA complexes was evaluated by agarose gel electrophoresis. Figure 1 shows that 9rR-LTVSPWY was able to bind DNA in a manner that depended on the N/P ratio and yielded complete retardation of the DNA at an N/P ratio ≥ 3, indicating that 9rR-LTVSPWY encapsulated the DNA completely at those N/P ratios. The N/P ratios represent the molar ratio of the arginines in 9rR (or the arginines in the nona-arginine fragment of the 9rR-LTVSPWY) to the phosphorus content in the DNA [19].

**Figure 2:**
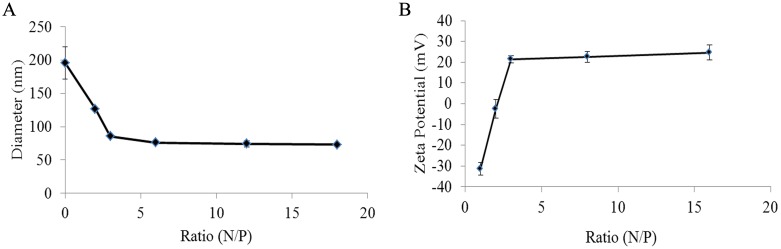
Characterization of the 9rR-LTVSPWY/pEGFP-N1 complexes at the indicated N/P ratios. (A) Particle size, (B) zeta potential (n = 3, mean ± SD). The polydispersity index of each sample was below 0.3.

Figure 2A shows the particle size values of the 9rR-LTVSPWY/pDNA complexes at various N/P ratios measured using dynamic light scattering (DLS). At an N/P ratio of 2, the particle sizes of the 9rR-LTVSPWY/pDNA mixture were ∼126 nm, which was smaller than that of the naked pDNA (195±24 nm) and larger than that of the mixture at N/P ratios ≥ 3; this demonstrated that the pDNA was partly condensed by the peptide. When the N/P ratio rose above 3, the particle sizes of the 9rR-LTVSPWY/pDNA complexes were decreased to approximately 75 nm, suggesting that the pDNA was efficiently encapsulated and condensed by 9rR-LTVSPWY. Figure 2B indicates that the zeta potential of the 9rR-LTVSPWY/pDNA mixture was negative at N/P ratio ≤ 2; at higher N/P ratios, the zeta potential of the 9rR-LTVSPWY/pDNA complexes turned positive. When the N/P ratio rose above 3, the zeta potential of the complexes was increased to approximately 21 mV. The particle size and zeta potential measurement results were consistent with the agarose gel electrophoresis assay and suggested that, at N/P ratios ≥ 3, 9rR-LTVSPWY encapsulates the DNA completely.

### Gene transfection into cells using 9rR-LTVSPWY/pDNA complexes

**Figure 3:**
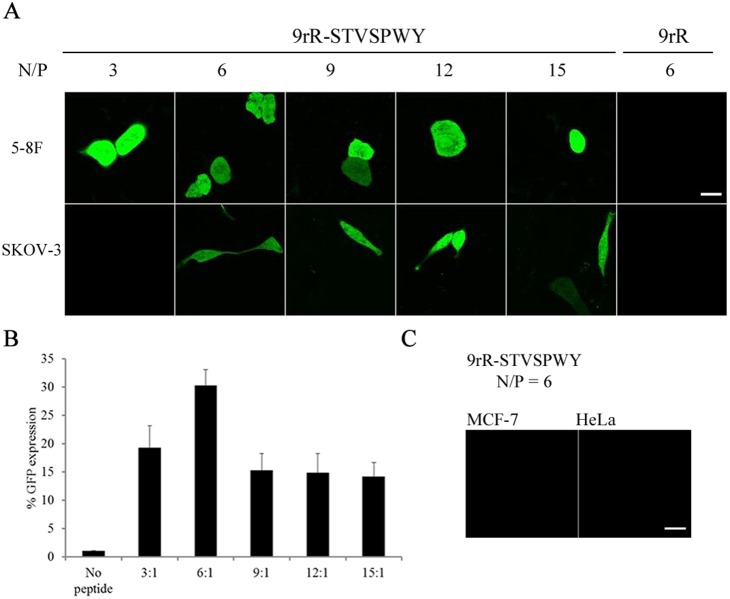
Cellular uptakes of 9rR-LTVSPWY at different N/P ratios in different cells. (A) Confocal microscopy images of 5-8F and SKOV-3 cells at 48 h after transfection with the 9rR-LTVSPWY/pEGFP-N1 or 9rR/pEGFP-N1 complexes at the indicated N/P ratios. (B) Percentage of 5-8F cells expressing GFP at 48 h after transfection with the 9rR-LTVSPWY/pEGFP-N1 complexes at the indicated N/P ratios or with a vehicle control (no peptide). Cellular fluorescence was examined by FACS. The data are shown as the means ± SD. All measurements were performed in triplicate. (C) Confocal microscopy images of MCF-7 and HeLa cells at 48 h after transfection with the 9rR-LTVSPWY/pEGFP-N1 complexes at an N/P ratio of 6. Green fluorescence represents pEGFP-N1 expression. (Scale bar = 20 µm).

Because the LTVSPWY peptide has been used as a specific ligand for the cell lines 5–8F and SKOV-3 [23], [27], these two cell lines were used to evaluate the ability of the 9rR-LTVSPWY/pDNA complexes to mediate DNA transfection, while MCF-7 and HeLa cells were used as negative controls. To optimize the peptide/pDNA stoichiometry (N/P ratio) for the best transfection efficiency, the transfection efficiencies of the 9rR-LTVSPWY/pEGFP-N1 complexes at various N/P ratios were measured using GFP as a reporter protein. Confocal microscopy (Figure 3A) showed significant pDNA expression (green fluorescence) in 5–8F and SKOV-3 cells transfected with 9rR-LTVSPWY/pEGFP-N1 at N/P ratios of 6, 9, 12 and 15. N/P = 6 yielded the highest number of cells expressing GFP (Figures 3A, 3B), suggesting that N/P = 6 is the optimal condition for transfection. In contrast, no pDNA expression was observed in MCF-7 and HeLa cells under the same conditions (Figure 3C), demonstrating the cell selectivity of the peptide/pDNA complexes. In contrast, although 9rR encapsulated DNA completely at N/P ratios ≥ 3 [24], there was no obvious GFP expression in the 5–8F cells transfected with the 9rR/pEGFP-N1 complex (Figure 3A and Figure S3), suggesting that the LTVSPWY sequence is essential for the cellular uptake of the 9rR-LTVSPWY/pDNA complexes.

**Figure 4:**
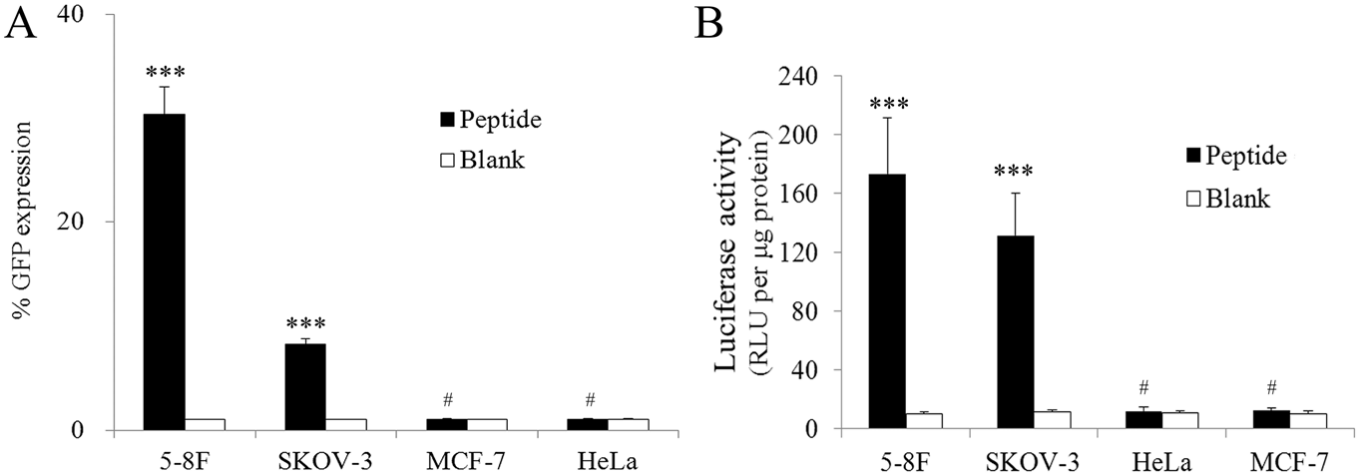
Comparison of the cellular uptakes of 9rR-LTVSPWY in different cells. (A) Percentage of 5-8F, SKOV-3, MCF-7 and HeLa cells expressing GFP at 48 h after transfection with the 9rR-LTVSPWY/pEGFP-N1 complexes (N/P 6:1, black bars) or a vehicle control (blank, white bars). (B) Quantitative evaluation of luciferase expression in 5-8F, SKOV-3, MCF-7 and HeLa cells at 48 h after transfection with the 9rR-LTVSPWY/pGL3 complex (N/P 6:1, black bars) or a vehicle control (blank, white bars). The data are shown as the means ± SD. All measurements were performed in triplicate. The “***” symbol indicates a significant difference between the two groups based on three independent experiments (p<0.001). The “#” symbol indicates no significant difference between the two groups based on three independent experiments (p>0.05).

Because the confocal imaging and FACS results suggested that N/P = 6 results in the highest transfection efficiency, we conducted further experiments with the peptide/pDNA complexes prepared at an N/P ratio of 6. To quantify the specificity and efficiency of transfection in various types of cells, we performed quantitative analysis using flow cytometry. Figure 4A showed that the percentages of 5–8F, SKOV-3, MCF-7 and HeLa cells expressing GFP were approximately 30.33, 8.23, 1.04 and 1.05, respectively, implying that the plasmids were expressed in 5–8F and SKOV-3 cells, with significantly different expression levels in the experimental groups and the blank group, but not to MCF-7 or HeLa, which exhibited no significant difference between the experimental groups and the blank group. Next, we performed luciferase expression assays using the four cell lines. Figure 4B indicates that 9rR-LTVSPWY delivered the plasmid into 5–8F and SKOV-3 cells but not MCF-7 or HeLa cells, verifying the excellent selectivity of this method.

### The cellular uptake of the 9rR-LTVSPWY/pDNA complex

**Figure 5:**
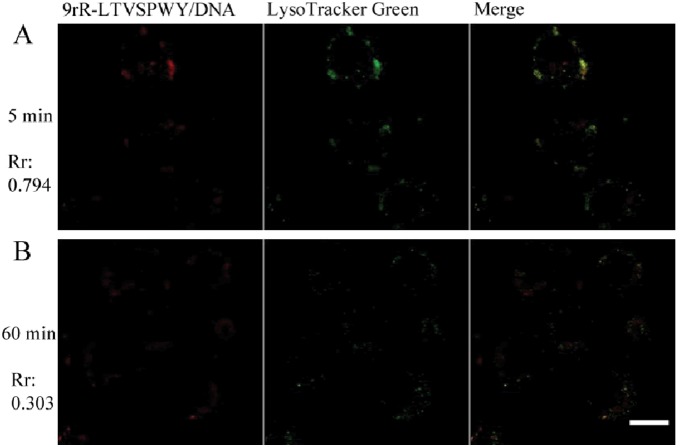
Confocal microscopy analysis of the intercellular distributions of 9rR-LTVSPWY/plasmid complexes. Living 5-8F cells were incubated with the 9rR-LTVSPWY/EMA-labeled pEGFP-N1 complex (N/P 6:1) and LysoTracker Green (100 nM) for 30 min at 37°C and post-incubation in RPMI1640 containing 10% FBS for (A) 5 min and (B) 60 min. Rr: Pearson’s coefficient, Red: EMA-labeled pDNA, green: LysoTracker Green, yellow: merge. (Scale bar = 10 µm).

**Figure 6:**
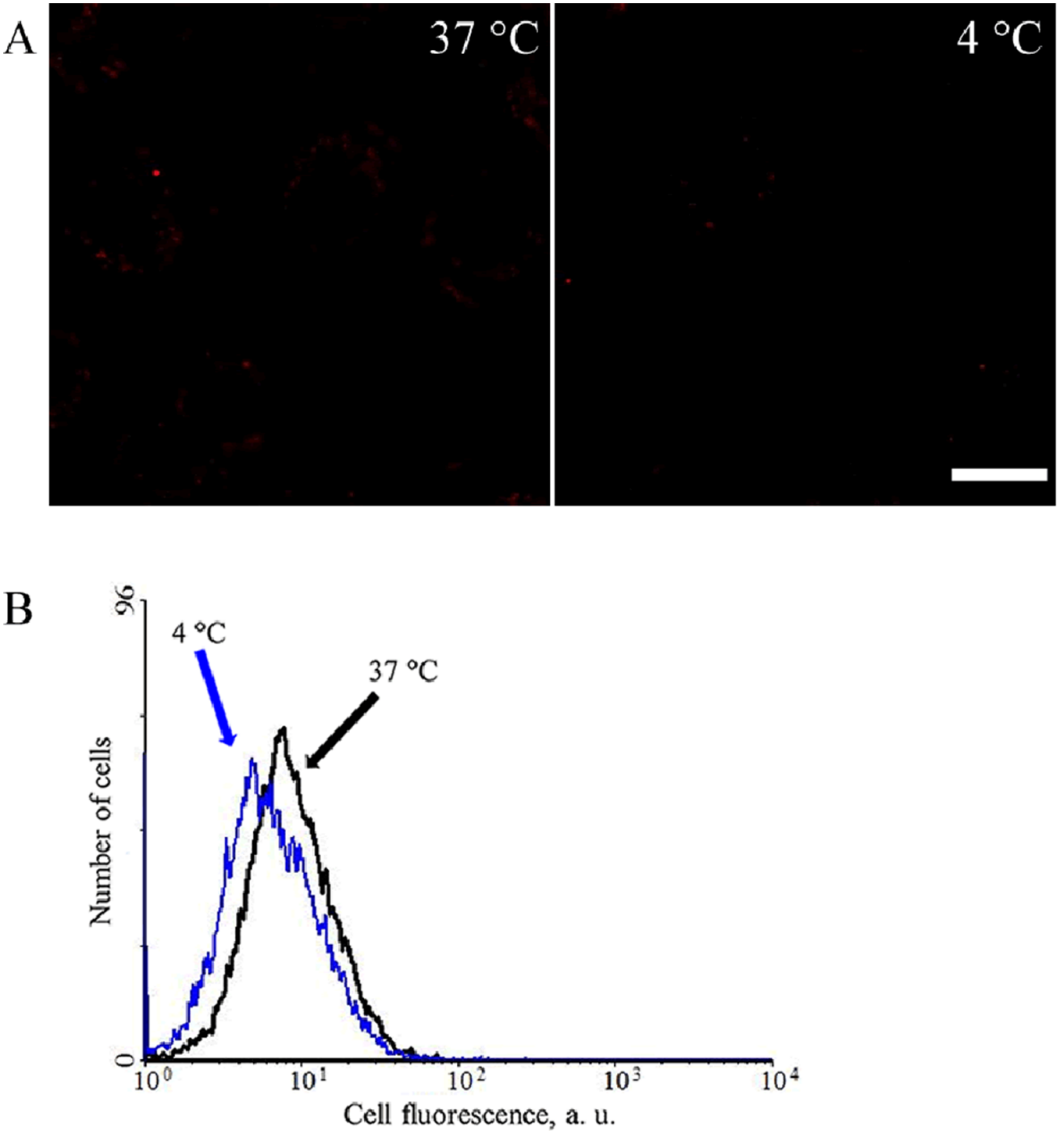
Comparison of the cellular uptakes of 9rR-LTVSPWY/plasmid complexes at different temperatures. Living 5-8F cells were incubated with the 9rR-LTVSPWY/EMA-labeled pEGFP-N1 complex (N/P 6:1) for 30 min at 37°C or 4°C and then examined by (A) Confocal microscopy or (B) flow cytometry analyses. (Scale bar = 10 µm).

The cellular uptake mechanism of the 9rR-LTVSPWY/pDNA complex was then investigated. 5–8F cells were incubated with EMA-labeled pEGFP-N1 (red) encapsulated with 9rR-LTVSPWY or 9rR. Figure 5A reveals that the EMA-labeled 9rR-LTVSPWY/pDNA complexes (red) were obviously absorbed by the cells in 30 min after incubation and mainly distributed in dots. These puncta co-localized well with the LysoTracker Green probe (green), with a Pearson’s coefficient of 0.794, suggesting that the complexes were mainly located in endosomes and/or lysosomes. In contrast, the 9rR/pDNA complex could not enter the cells at all (Figure S3). When the incubation temperature was decreased from 37°C to 4°C, which typically inhibits endocytosis, the cellular uptake of the 9rR-LTVSPWY/pDNA complex was significantly decreased (Figure 6). These results suggest that the 9rR-LTVSPWY/pDNA complex entered the cells via receptor-mediated endocytosis, consistent with the previously reported cellular uptake mechanism of LTVSPWY [27]. One hour post-incubation, the pDNA (red fluorescence) had escaped from the endosomes (green fluorescence) and was diffusely distributed in the cytosol (Figure 5B), with a resulting decrease in the Pearson’s coefficient (to 0.303). The results showed that pDNA transfected into cells using 9rR-LTVSPWY can escape from endocytic vesicles quickly.

### Cytotoxicity of the 9rR-LTVSPWY/pDNA complex in 5–8F cells

A MTT assay was conducted to measure the cytotoxicity of the 9rR-LTVSPWY/pDNA complex in 5–8F cells. Our result (Figure S4) illustrated that the viability of the cells incubated with 9rR-LTVSPWY/pDNA complexes was approximately 93%, suggesting that the cytotoxicity of the 9rR-LTVSPWY/pDNA complex is low.

## Discussion

The development of tumor-targeted DNA therapeutics is still hindered by the lack of efficient, selective and safe DNA delivery systems. Although viral vectors have high transfection efficiencies, they present serious safety issues in therapeutic applications. Because of this concern, non-viral vectors have been developed as safer alternatives for clinical applications. Of the non-viral vectors, cationic lipids or polymers are frequently used due to several advantages, such as their low immunogenicity and ease of use. However, their toxicity and transfection efficiency problems still limit the tumor-targeting DNA delivery applications.

Compared with lipid- or polymer-based DNA delivery vectors, peptide-based DNA delivery systems are more biocompatible and biodegradable and therefore have lower immunogenicity and lower toxicity. Moreover, peptides are easier to design and produce because solid-phase peptide synthesis has been extensively developed. Thus, the development of peptides that can deliver DNA specifically to cancer cells is very relevant for cancer gene therapy.

In this study, we tested a peptide-based selective DNA delivery system in specific tumor cells. We demonstrated that at N/P ratios ≥ 3, the 9rR-LTVSPWY peptide efficiently condensed extended pDNA (∼195 nm in diameter), leading to the formation of compacted nanoparticles of ∼75 nm in diameter with a positive charge (∼ 21 mV) (Figure 3). Compared to the particle size of polymer/pDNA (> 100 nm) or liposome/pDNA complexes (> 100 nm) [28], the ∼75 nm diameter of the 9rR-LTVSPWY/pDNA is much smaller, which may lend the particle greater efficiency and penetration into tumor tissue [29]. This size is also large enough to avoid rapid renal clearance [30]. Certain types of nanoparticles work less effectively due to aggregation in the blood. Aggregation of 9rR-LTVSPWY/pDNA complexes is avoided by their positive charge, which exerts an electrostatic repulsion force [31], [32].

LTVSPWY can bind specifically to certain tumor cells and be absorbed via receptor-mediated endocytosis. Our results showed that 9rR-LTVSPWY successfully delivered pDNA into these tumor cells. The peptide/pDNA stoichiometry (N/P ratio) was optimized to obtain the highest transfection efficiency, which was then achieved with an N/P ratio of 6 (Figure 3). Quantitative analysis based on FACS and luciferase expression revealed that 9rR-LTVSPWY can deliver DNA into 5–8F cells and SKOV-3 cells but not into receptor-negative MCF-7 cells or HeLa cells (Figures 4), suggesting that 9rR-LTVSPWY successfully mediates cell-specific DNA delivery. By altering the receptor sequence, more peptide-based DNA delivery systems can be developed for various types of cancers.

The results of our experiments studying the cellular uptake and intracellular distribution of the peptide/pDNA complexes (Figure 5 and 6) suggested that the LTVSPWY-9rR/pDNA complex enters the cells via endocytosis, like LTVSPWY alone. The findings that 5–8F cells could not take up the 9rR/pDNA complex and that 9rR-LTVSPWY could not deliver DNA into receptor-negative MCF-7 cells or HeLa cells (Figure 3, 4, and S3) imply that the cellular uptake of the 9rR-LTVSPWY/pDNA complex is mediated by an LTVSPWY receptor. The significant intracellular uptake of the 9rR-LTVSPWY/pDNA complex observed within 30 min of treatment shows that this endocytosis process was fairly rapid, most likely because the electrostatic interactions between the positive charge (∼21 mV) of the complex and the negatively charged cell membrane enhanced the effective local concentration of the complex at the cell surface, increasing the efficiency of cellular uptake [33]. Release from endocytic vesicles is one of the prerequisites for efficient DNA therapy [26]. The pDNA/9rR-LTVSPWY complex escaped from the endocytic vesicles quickly (within 1.5 h). This effect was most likely due to proton scavenging effects of the histidine residues in the 9rR-LTVSPWY sequence, which can disrupt endocytic vesicles [10], and to fusion effects of the arginine residues (9rR), which can enhance endosomal escape through liposomal fusion to the endosomal membrane at both acidic and neutral pH [26], [34]. These results may aid in the designing of a more efficient DNA delivery system.

In summary, a non-viral 9rR-LTVSPWY peptide-based DNA delivery system was constructed that can mediate efficient DNA expression in only selected tumor cells. The advantages of this system include its ease of synthesis, low immunogenicity, biocompatibility, low cytotoxicity and selectivity. The results suggest a strategy for tumor cell-specific DNA delivery and provide a reference for designing more efficient DNA delivery systems targeted towards various types of cancers. However, for *in* *vivo* applications, the systemic toxicity, the *in* *vivo* dose, and the *in* *vivo* transfection efficiency of 9rR-LTVSPWY DNA delivery system need further careful evaluated. *In vivo* studies on our system are currently underway.

## Supporting Information

Figure S1 HPLC analysis of 9rR-LTVSPWY.
(PDF)

Figure S2 ESI-MS analysis of 9rR-LTVSPWY.
(PDF)

Figure S3 Confocal microscopy images of 5–8F cells after incubation with the 9rR/EMA-labeled pDNA complex (N/P 6∶1) for 30 min at 37°C. (Scale bar = 10 µm).
(TIF)

Figure S4 Cell viability of 5-8F cells at 48 h after transfection with the 9rR-LTVSPWY/pEGFP-N1 complex (N/P 6:1). The data are shown as the means ± SD.
(TIF)

## References

[1] Münch RC, Janicki H, Völker I, Rasbach A, Hallek M, et al (2013) Displaying High-affinity Ligands on Adeno-associated Viral Vectors Enables Tumor Cell-specific and Safe Gene Transfer. Mol Ther 21: 109–118. 22968478 10.1038/mt.2012.186 PMC3538307

[2] Naldini L (2001) Viral vectors for gene therapy: the art of turning infectious agents into vehicles of therapeutics. Nat Med 7: 33–39. 11135613 10.1038/83324

[3] Huang S, Kamihira M (2012) Development of hybrid viral vectors for gene therapy. Biotechnol Adv 31: 208–223. 23070017 10.1016/j.biotechadv.2012.10.001

[4] He H, Kong SK, Chan KT (2011) Transfection and cell fusion by femtosecond lasers. J Innov Opt Health Sci 04: 113–125.

[5] Lehrman S (1999) Virus treatment questioned after gene therapy death. Nature 401: 517–518. 10524611 10.1038/43977

[6] Kim PH, Kim T, Yockman JW, Kim SW, Yun CO (2010) The effect of surface modification of adenovirus with an arginine-grafted bioreducible polymer on transduction efficiency and immunogenicity in cancer gene therapy. Biomaterials 31: 1865–1874. 19962189 10.1016/j.biomaterials.2009.11.043

[7] Lv H, Zhang S, Wang B, Cui S, Yan J (2006) Toxicity of cationic lipids and cationic polymers in gene delivery. J Control Release 114: 100–109. 16831482 10.1016/j.jconrel.2006.04.014

[8] Zhang Y, Satterlee A, Huang L (2012) In Vivo Gene Delivery by Nonviral Vectors: Overcoming Hurdles&quest. Mol Ther 20: 1298–1304. 22525514 10.1038/mt.2012.79 PMC3392980

[9] Martin ME, Rice KG (2007) Peptide-guided gene delivery. AAPS J 9: 18–29. 10.1208/aapsj0901003 PMC2751301 17408236

[10] Seow WY, Yang YY, George AJT (2009) Oligopeptide-mediated gene transfer into mouse corneal endothelial cells: expression, design optimization, uptake mechanism and nuclear localization. Nucleic Acids Res 37: 6276–6289. 19692581 10.1093/nar/gkp651 PMC2764440

[11] Sadatmousavi P, Soltani M, Nazarian R, Jafari M, Chen P (2011) Self-assembling peptides: potential role in tumor targeting. Curr Pharm Biotechnol 12: 1089–1100. 21470142 10.2174/138920111796117409

[12] Standley SM, Toft DJ, Cheng H, Soukasene S, Chen J, et al (2010) Induction of cancer cell death by self-assembling nanostructures incorporating a cytotoxic peptide. Cancer Res 70: 3020–3026. 20354185 10.1158/0008-5472.CAN-09-3267 PMC2893556

[13] Ruoslahti E (2012) Peptides as targeting elements and tissue penetration devices for nanoparticles. Adv Mater 24: 3747–3756. 22550056 10.1002/adma.201200454 PMC3947925

[14] Fonseca SB, Pereira MP, Kelley SO (2009) Recent advances in the use of cell-penetrating peptides for medical and biological applications. Adv Drug Deliver Rev 61: 953–964. 10.1016/j.addr.2009.06.001 19538995

[15] Nakase I, Akita H, Kogure K, Graslund A, Langel Ul, et al (2012) Efficient intracellular delivery of nucleic acid pharmaceuticals using cell-penetrating peptides. Account Chem Res 45: 1132–1139. 10.1021/ar200256e 22208383

[16] Wender PA, Galliher WC, Goun EA, Jones LR, Pillow TH (2008) The design of guanidinium-rich transporters and their internalization mechanisms. Adv Drug Deliver Rev 60: 452–472. 10.1016/j.addr.2007.10.016 PMC2533582 18164781

[17] Wang HY, Chen JX, Sun YX, Deng JZ, Li C, et al (2010) Construction of cell penetrating peptide vectors with N-terminal stearylated nuclear localization signal for targeted delivery of DNA into the cell nuclei. J Control Release 155: 26–33. 21187118 10.1016/j.jconrel.2010.12.009

[18] Khalil I, Futaki S, Niwa M, Baba Y, Kaji N, et al (2004) Mechanism of improved gene transfer by the N-terminal stearylation of octaarginine: enhanced cellular association by hydrophobic core formation. Gene Ther 11: 636–644. 14973542 10.1038/sj.gt.3302128

[19] Seow WY, Yang YY (2009) A Class of Cationic Triblock Amphiphilic Oligopeptides as Efficient Gene-Delivery Vectors. Adv Mater 21: 86–90.

[20] Liu L, Zheng M, Renette T, Kissel T (2012) Modular Synthesis of Folate Conjugated Ternary Copolymers: Polyethylenimine-graft-Polycaprolactone-block-Poly (ethylene glycol)-Folate for Targeted Gene Delivery. Bioconjugate Chem 23: 1211–1220. 10.1021/bc300025d 22548308

[21] Minko T (2012) Receptor Mediated Delivery Systems for Cancer Therapeutics. Fundamentals and Applications of Controlled Release Drug Delivery. US: Springer 329–355 p.

[22] Shadidi M, Sioud M (2003) Identification of novel carrier peptides for the specific delivery of therapeutics into cancer cells. FASEB J 17: 256–258. 12490548 10.1096/fj.02-0280fje

[23] Luo H, Yang J, Jin H, Huang C, Fu J, et al (2011) Tetrameric far-red fluorescent protein as a scaffold to assemble an octavalent peptide nanoprobe for enhanced tumor targeting and intracellular uptake in vivo. FASEB J 25: 1865–1873. 21350116 10.1096/fj.10-174318

[24] Gong C, Li X, Xu L, Zhang YH (2012) Target delivery of a gene into the brain using the RVG29-oligoarginine peptide. Biomaterials 33: 3456–3463. 22322199 10.1016/j.biomaterials.2011.12.017

[25] Song L, Yan J, Jian S, Zhang L, Li M, et al (2002) Molecular mechanisms of tumorgenesis and metastasis in nasopharyngeal carcinoma cell sublines. Ai Zheng 21: 158–162. 12479066

[26] Ma Y, Gong C, Ma Y, Fan F, Luo M, et al (2012) Direct Cytosolic Delivery of Cargoes in vivo by a Chimera Consisting of D-and L-Arginine Residues. J Control Release 162: 286–294. 22824782 10.1016/j.jconrel.2012.07.022

[27] Wang XF, Birringer M, Dong LF, Veprek P, Low P, et al (2007) A peptide conjugate of vitamin E succinate targets breast cancer cells with high ErbB2 expression. Cancer Res 67: 3337–3344. 17409443 10.1158/0008-5472.CAN-06-2480

[28] Won YW, Kim HA, Lee M, Kim YH (2009) Reducible poly (oligo-D-arginine) for enhanced gene expression in mouse lung by intratracheal injection. Mol Ther 18: 734–742. 20029398 10.1038/mt.2009.297 PMC2862522

[29] Jiang W, Kim BYS, Rutka JT, Chan WCW (2008) Nanoparticle-mediated cellular response is size-dependent. Nat Nanotechnol 3: 145–150. 18654486 10.1038/nnano.2008.30

[30] Choi HS, Liu W, Misra P, Tanaka E, Zimmer JP, et al (2007) Renal clearance of quantum dots. Nat Biotechnol 25: 1165–1170. 17891134 10.1038/nbt1340 PMC2702539

[31] Wang S, Song H, Ong WY, Han MY, Huang D (2009) Positively charged and pH self-buffering quantum dots for efficient cellular uptake by charge mediation and monitoring cell membrane permeability. Nanotechnology 20: 425102–425109. 19779229 10.1088/0957-4484/20/42/425102

[32] Chi EY, Krishnan S, Randolph TW, Carpenter JF (2003) Physical stability of proteins in aqueous solution: mechanism and driving forces in nonnative protein aggregation. Pharm Res 20: 1325–1336. 14567625 10.1023/a:1025771421906

[33] Henriques ST, Melo MN, Castanho MARB (2006) Cell-penetrating peptides and antimicrobial peptides: how different are they? BioChem J 399: 1–7. 16956326 10.1042/BJ20061100 PMC1570158

[34] El-Sayed A, Khalil IA, Kogure K, Futaki S, Harashima H (2008) Octaarginine-and octalysine-modified nanoparticles have different modes of endosomal escape. J Biol Chem 283: 23450–23461. 18550548 10.1074/jbc.M709387200

