# Supplementary figures and images for: Selective DNA Delivery to Tumor Cells Using an Oligoarginine-LTVSPWY Peptide
Source: PLoS One. 2014 Oct 22;9(10):e110632. doi: 10.1371/journal.pone.0110632 (PMC4206439; doi:10.1371/journal.pone.0110632)

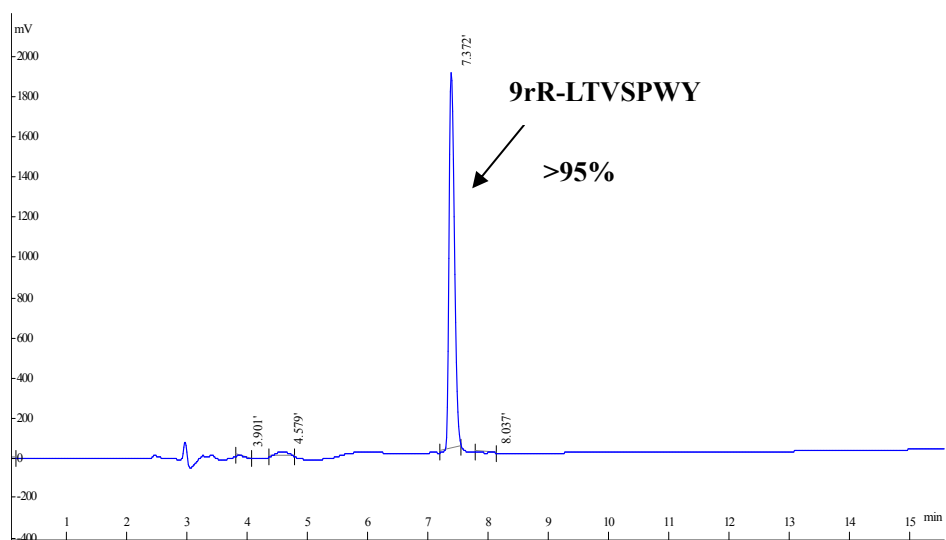

| Rank  | Time   | Conc     | Area     |
|-------|--------|----------|----------|
| 1     | 0.094  | 0.002996 | 366      |
| 2     | 3.901  | 0.4812   | 58774    |
| 3     | 4.579  | 3.383    | 413175   |
| 4     | 7.372  | 96.14    | 11743067 |
| 5     | 10.406 | 0        | 2867079  |
| Total |        | 100      | 12187446 |

Supplement: Figure S1 — HPLC analysis of 9rR-LTVSPWY. (PDF) [file pone.0110632.s001.pdf]

24-May-2010  
17:08:06  
MW:2819.27  
100524-YH-1 14 (0.959)

Probe: ESI  
Cone: 50v  
Desolvation Temp :350

Capillary:3.00KV  
Extractor: 5v  
Gas Flow : 350  
Scan ES+  
1.42e7

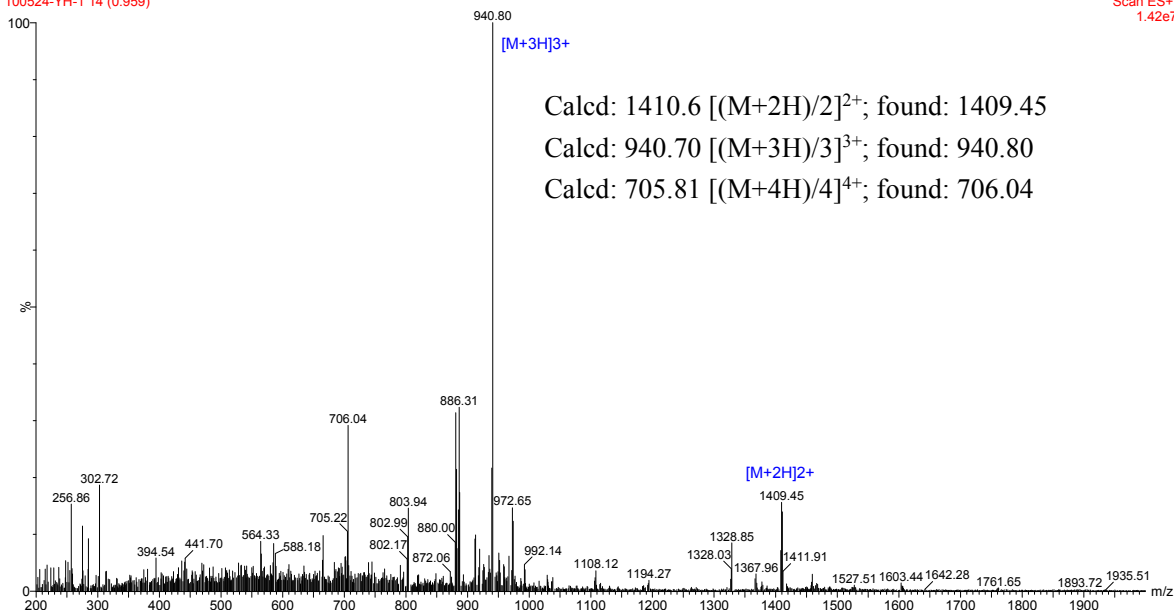

Supplement: Figure S2 — ESI-MS analysis of 9rR-LTVSPWY. (PDF) [file pone.0110632.s002.pdf]

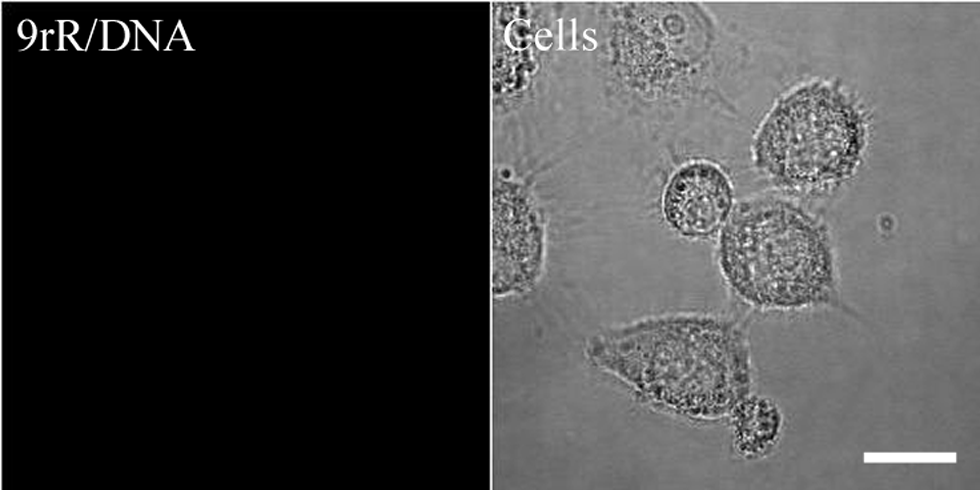

Supplement: Figure S3 — Confocal microscopy images of 5–8F cells after incubation with the 9rR/EMA-labeled pDNA complex (N/P 6∶1) for 30 min at 37°C. (Scale bar = 10 µm). (TIF) [file pone.0110632.s003.tif]

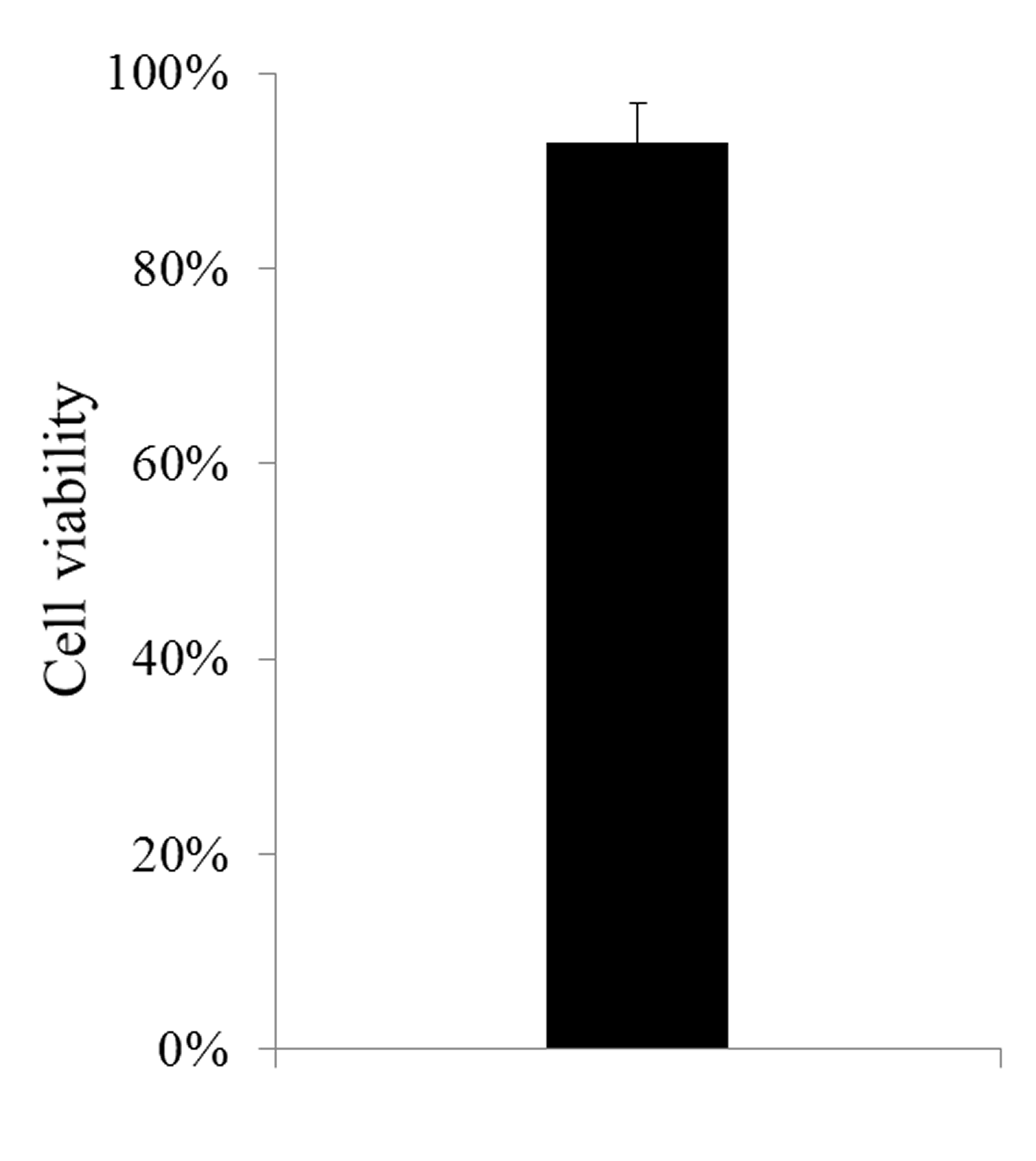

Supplement: Figure S4 — Cell viability of 5-8F cells at 48 h after transfection with the 9rR-LTVSPWY/pEGFP-N1 complex (N/P 6:1). The data are shown as the means ± SD. (TIF) [file pone.0110632.s004.tif]
